# Using *Scipion* for stream image processing at Cryo-EM facilities

**DOI:** 10.1016/j.jsb.2018.10.001

**Published:** 2018-12

**Authors:** J. Gómez-Blanco, J.M. de la Rosa-Trevín, R. Marabini, L. del Cano, A. Jiménez, M. Martínez, R. Melero, T. Majtner, D. Maluenda, J. Mota, Y. Rancel, E Ramírez-Aportela, J.L. Vilas, M. Carroni, S. Fleischmann, E. Lindahl, A.W. Ashton, M. Basham, D.K. Clare, K. Savage, C.A. Siebert, G.G. Sharov, C.O.S. Sorzano, P. Conesa, J.M. Carazo

**Affiliations:** a Department of Anatomy and Cell Biology, McGill University, Montreal, Canada; b Biocomputing Unit, National Center for Biotechnology (CSIC), C/ Darwin, 3, Campus Universidad Autónoma, 28049 Cantoblanco, Madrid, Spain; c Department of Biochemistry and Biophysics, Science for Life Laboratory, Stockholm University, Stockholm, Sweden; d Escuela Politécnica Superior, Universidad Autónoma de Madrid, 28049 Cantoblanco, Madrid, Spain; e Swedish e-Science Research Center, KTH Royal Institute of Technology, Stockholm, Sweden; f Diamond Light Source, Harwell Science and Innovation Campus, Didcot OX11 0DE, United Kingdom; g Medical Research Council Laboratory of Molecular Biology, Francis Crick Avenue, Cambridge CB2 OQH, United Kingdom

**Keywords:** Electron microscopy, Streaming, Image processing, Live processing, High throughput, *Scipion*

## Abstract

Three dimensional electron microscopy is becoming a very data-intensive field in which vast amounts of experimental images are acquired at high speed. To manage such large-scale projects, we had previously developed a modular workflow system called *Scipion* (de la Rosa-Trevín et al., 2016). We present here a major extension of *Scipion* that allows processing of EM images while the data is being acquired. This approach helps to detect problems at early stages, saves computing time and provides users with a detailed evaluation of the data quality before the acquisition is finished. At present, *Scipion* has been deployed and is in production mode in seven Cryo-EM facilities throughout the world.

## 1 Introduction

3D electron microscopy (3DEM) can provide rich information about structural characteristics of macromolecular complexes. The field is under a major transformation due to the arrival of better microscopes, new large area direct electron detectors and automation (Kuhlbrandt, 2014a, Kuhlbrandt, 2014b). These improvements make possible that a single microscope generates high quality data sets on the order of terabytes per day (Saibil et al., 2015) while working for several days without interruption. Major challenges faced in the field are: (1) an efficient management of these huge datasets and their corresponding image processing workflows; (2) the need for sharing data and metadata in an increasingly distributed and collaborative environment and; (3) a raising interest from scientists outside the field who might lack the skills of a experienced microscopist.

For most cases, the future of projects that require high-end microscopes is through centralized microscopy facilities, in which automatically acquired data needs to be monitored by users and facility staff. With this scenario in mind, we have redesigned *Scipion* which is a software framework for integrating several 3DEM software packages through a workflow-based approach (de la Rosa-Trevín et al., 2016). In this way, *Scipion* starts the processing of the movies as they are being acquired with the double aim of checking for possible data collection errors and executing a user-tailored image processing workflow that provides users with an accurate estimation of the acquired data quality.

Many software packages have been developed to support automated data collection, such as SerialEM (Mastronarde, 2005), Leginon (Suloway et al., 2009), UCSF-Tomography (Zheng et al., 2007), Tom Toolbox (Nickell et al., 2005), EPU (Thermo Fisher Scientific, 2018), Latitude in Digital Micrograph (Gatan, Inc, 2018), EMMenu (Tietz, GmbH, 2018), etc. However, the next step, which is to provide image processing while data are being collected, is not so well addressed. In fact, in most places this is accomplished through home made scripts (e.g. Pichkur et al., 2018). Exceptions are UCSFImage4 (Li et al., 2015), Focus (Biyani et al., 2017), Warp (Tegunov and Cramer, 2018) and Relion (Fernandez-Leiro and Scheres, 2017). As we will show in this article, *Scipion* differs from these solutions in its larger flexibility, both from the point of view of users and facility staff.

The organization of this document is as follows. First, we present the modifications introduced in *Scipion* to adapt it to large centralized microscopy facilities. Then we present a typical workflow to demostrate *Scipion* capabilities. Finally, we describe in detail how *Scipion* is being used in three representative facilities.

## 2 Main Modifications introduced in *Scipion*: Streaming and Metaprotocols

In the following we describe the most important modifications made to *Scipion* to facilitate its deployment and execution in centralized facilities. The section ends with a very brief description of *Scipion* design and architecture that highlights the most interesting features of this image processing framework.

### 2.1 Stream processing

Stream processing, i.e., computing on data directly as they are produced or received, is the main change introduced in *Scipion*. In this way, a new level of parallelization has been reached since the data generated by the microscope continuously flows through a chain of protocols. For example, as movies are imported to the system they are aligned and their CTF is estimated. In *Scipion* version 1.2, users may find the first steps in the image processing workflow adapted to streaming, including movie alignment, CTF estimation and particle picking and extraction. In the developers version, several 2D classification algorithms are being adapted for this new way of processing.

*Scipion* stream implementation make extensive use of threads. Using threads, a programmer can effectively create two or more tasks that run at the same time and share the protocol resources (e.g. memory). Each protocol that works in streaming mode has one of its threads monitoring input and updating output data while other threads take care of the data processing. This clear separation between data access and data processing marks an important difference between running a script or using *Scipion*, since it facilitates a form of computing in which several jobs are executed concurrently, instead of sequentially.

### 2.2 Metaprotocols

Since the *Scipion* original publication we have developed many *metaprotocols*, that is, protocols that either check the progress of other protocols or compare the results of equivalent protocols.

The first type of metaprotocols are called *monitors* and are used to produce live analysis plots, generate reports or raise alerts when some problems are detected. A monitor example is the CTF-monitor, that checks the computed defocus values for each micrograph as they are generated. CTF-monitor may raise an alert if the defocus values are above or below certain thresholds and continuously generate HTML files so that users and staff may easily follow the data acquisition and processing either in-house or remotely. An example of these reports may be seen at  http://scipion.cnb.csic.es/scipionbox/lastHTMLReport/.

The second set of metaprotocols are grouped under the name *consensus*. For a given logical step (for example, particle picking) these protocols check the consistency of the output datasets obtained from the same input data using different algorithms. Continuing with the particle picking example, consensus picking will compare, at a given instant, the particles selected for each one of the executed particle-picking algorithms and produce as output a particle set containing those particles selected by most of the algorithms.

### 2.3 *Scipion* design and architecture

We would like to finish this section commenting on some of *Scipion* design decisions that differentiate *Scipion* from other image processing frameworks. *Scipion* is fully oriented to integrate existing EM software and therefore the versatility and number of possible steps combination is huge. Additionally, *Scipion* takes care of keeping a rigorous traceability and reproducibility as well as to provide an API (application programming interface) to allow the integration with already existing LIMS systems. At the software architectural level, the greatest difference between *Scipion* and the other EM image processing packages mentioned in the introduction is that *Scipion* pays special attention to define abstraction layers to simplify maintenance and extensibility and follows a strict workflow approach. A few examples will illustrate the main advantages of this approach.

To create a new protocol in *Scipion*, a single *Python* file needs to be created. From this file, the system will discover the new protocol and will automatically build a form and store the protocol related information in a database. *Scipion* protocol developers must certainly know *Scipion* conventions but they do not need to modify or even understand the existing code. In *Scipion* next version, the abstraction will be increased since protocols will be implemented as plug-ins, that is, independent small *Python* files that add functionality to the main *Scipion* application. The plug-in approach allows parallel development. That is, since protocols are implemented independently from the main application they can be developed and released in parallel by different teams. In this way, support for a new version of *Relion* does not require a new *Scipion* release but a release of the independent *Relion* plug-in.

*Scipion* follows a pure workflow approach and therefore, implements pipelines and describes them as a set of interconnected steps (or protocols). In a workflow approach, users (including microscope staff) can reasonably be expected to modify the paths and incorporate new steps using a GUI (graphic user interface). On the contrary, software packages that do not make this separation produce workflows that, in general, are more challenging to create or modify for non programmers.

Another advantage of the workflow approach is the improvement of load balance, reproducibility and provenance (process of tracing and recording the origins of data and its movement between protocols). The first step in a *Scipion* workflow execution is to store in a database each of the protocols to be executed as well as how outputs of one protocol are attached to the inputs of another protocol. Then, a workflow engine analyzes the stored data and, using as constraint the number of CPUs assigned by the user to the workflow, optimizes the execution, that is, tries to execute as many protocols in parallel as possible. Obviously, the data stored in the database have enough information to execute each step and therefore guarantee reproducibility and provenance. Additionally, *Scipion* currently exports a JSON (JavaScript Object Notation) file describing the complete preprocessing workflow used, so that it can be fully reproduced and visualized elsewhere.

## 3 Image processing workflows

*Scipion* is a flexible framework that allows to create different workflows by choosing among many algorithms at each step. Although the best workflow for each case will depend on specific requirements of specimens, microscopes or facilities, we describe the pipeline offered by default at the *National Center for Biotechnology* (CNB) cryoEM Facility as use case to clarify *Scipion* capabilities.

The workflow starts two independent protocols in parallel. The first protocol estimates the camera gain at each pixel for a few movies (Sorzano et al., 2018). The output of this protocol serves as verification of the validity of the experimental gain image for the whole data set. The second protocol performs direct detector movie alignment. Currently, *Scipion* offers five methods for movie alignment: motioncorr (Li et al., 2015), motioncor2 (Zheng et al., 2016), correlation (an unpublished CPU version that implements in Xmipp a global alignment method and solves it similarly to motioncorr but using more robust statistics), unblur (Grant and Grigorieff, 2015) and optical flow (Abrishami et al., 2015). Then the CTFs of the resulting micrographs are calculated. *Scipion* provides three CTF estimation methods: CTFFIND4 (Rohou and Grigorieff, 2015), gCTF (Zhang, 2016) and Xmipp CTF estimation (Sorzano et al., 2007, Vargas et al., 2013). At this point the user may compute the CTFs using several algorithms and compare them with the protocol CTF consensus that reports up to which frequency two estimations of the same micrograph are equivalent. Further automatic filtering based on CTF is available through the protocol CTF filtering that selects micrographs based on the astigmatism, resolution and defocus.

Up to this point the image processing pipeline is quite solid and may be executed in streaming without user intervention. The next step is to select (pick) particles. We have recently adapted eight particle picking methods in *Scipion* for streaming (see at  https://github.com/I2PC/scipion/wiki/Integrated-Protocols#particles an exhaustive list). In this way, particles may be selected as soon as any new micrograph is available. Some picking algorithms require a training step in which either user intervention or information from a previous selection is needed. The streaming functionality is separated from the picking logic, so if in the future a new algorithm is added, it will work in streaming without extra effort from the algorithm developer. For those users that execute several picking algorithms, the protocol picking consensus may be useful. picking consensus calculates the agreement between different particle-picking algorithms and produces a list with the particles selected by most of them.

Two new streaming protocols are under development and will join the described pipeline in a near future. The first one will perform 2D classification and the second one will create an initial 3D map based on these classes. A first version of these protocols is available in the *Scipion* developers version.

As commented in Section 2, to monitor, chart and create alerts we have developed a set of metaprotocols. By default, system, CTF and report monitors are executed. System monitor reports on CPU and GPU usage, memory consumption, and disk I/O. CTF monitor plots the CTF defocus and astigmatism and it will raise an alarm if these parameters are not within a given range. Finally, the report protocol pushes all the created plots and summaries to a web server. This information can be accessed by users who are not physically in the microscope room (a live example report is available at  http://nolan.cnb.csic.es/scipionbox/lastHTMLReport/).

The proposed pipeline is very flexible and can be easily modified by each user. At the CNB (and collaborating institutions) we collect protocol usage statistics within *Scipion* and sort the different algorithms by popularity. This information is available at  http://scipion.i2pc.es/report_protocols/protocolTable/ and may help users to select between the different algorithms.

## 4 *Scipion* setup at different facilities

The data flow created by an electron microscope requires a careful design of the IT infrastructures. Different facilities have adopted different solutions and therefore, how *Scipion* access data and interchange information may require minor adjustments. In this section we discuss the solutions applied by some facilities in which *Scipion* is running.

So far *Scipion* is being used routinely in seven Cryo-EM facilities:

• *The Swedish National Cryo-EM Facility*. The Facility has two nodes, at SciLifeLab in Stockholm and at Umeå University ( http://www.scilifelab.se,  http://www.kbc.umu.se/english/ucem/cryo-em/)
• *ESRF Cryo-Electron Microscope* ( http://www.esrf.eu/home/UsersAndScience/Experiments/MX/About_our_beamlines/CM01.html)
• *eBIC at Diamond Light Source* ( http://www.diamond.ac.uk/Science/Integrated-facilities/eBIC.html)
• *CNB CryoEM Service* ( http://www.cnb.csic.es/index.php/en/research/core-facilities/microscopia-crioelectronica)
• *Molecular Microscopy Consortium – NIH* ( https://www.niehs.nih.gov/research/atniehs/facilities/mmc/index.cfm)
• *National Cancer Institute – NIH* ( https://www.cancer.gov/research/resources/cryoem)
• *University of Virginia Health System University Hospital* ( https://med.virginia.edu/molecular-electron-microscopy-core/)

*Scipion* original design satisfied CNB requirements, in which *Scipion* automatically fetches newly recorded movies from a network mounted disk. After this first installation, *Scipion* was deployed on SciLifeLab (a centralized facility located at Stockholm), where a rigorous booking system is followed. There, middleware (software that acts as a bridge between applications) was developed to interface with this booking platform to produce more precise reports. Lately, *Scipion* has been installed in large synchrotrons such as ESRF and Diamond, where projects are handled by ISPyB (a customized laboratory information management system -LIMS- (Delageniere et al., 2011)) and data are saved on a distributed file system. In this environment, *Scipion* needs to constantly interchange information with ISPyB and with other applications through a message queue system called *ActiveQ*.

In the following, through the three above mentioned use cases, we describe in detail how *Scipion* is been used in these different environments. For each use case we will describe first the facility setup and then in the subsection entitled “Aditional Software Developments” we will comment on specific software developed for each facility that connects *Scipion* with the facility and microscope control software.

### 4.1 Case 1: Scipion at CNB

The National Center for Biotechnology (CNB) forms part of the Spanish National Research Council (CSIC), the largest public research institution in Spain. The CNB Cryo-Electron Microscopy Facility is a joint effort of the Nacional Center for Biotechnology and the Biological Research Centers.

This section in divided in two parts. In the first one we discuss the network setup that allows us to move the data from the microscope to the user laboratory. Then, we comment on an additional software called *EMAdmin*. *EMAdmin* has been specifically designed for the CNB facility, it connects *Scipion* with the microscope acquisition software (*EPU*) and records the facility activity.

#### 4.1.1 Network setup and IT infrastructure

**Fig. 1:**
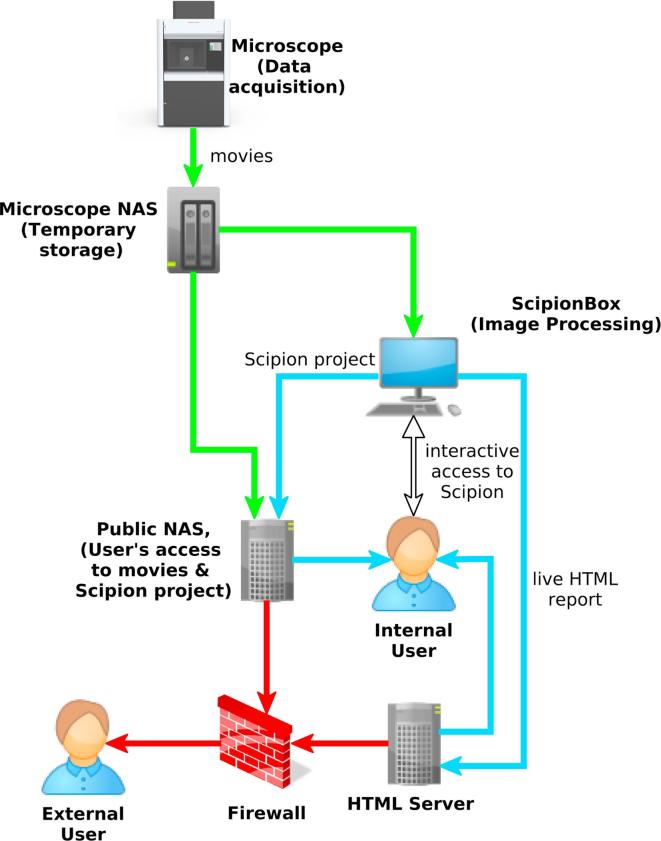
Graphical representation of the data flow through the CNB facility. Green, blue and red lines represent the microscope private network, the CNB local area network and the public (WAN) networks respectively.

At the beginning CNB networking setup was a quite straightforward one, movies were stored in the same machine that ran *Scipion* and processed data were saved to external USB hard disks by this same machine. The situation changed when the new Falcon-III detector was installed. After that, data were produced at sustained speeds as high as 90 MB/s (when acquiring four movies by hole in linear mode) and the main problem became to move smoothly the data through the entire pipeline from the microscope to the final user laboratory. In the following we describe such a pipeline (see Fig. 1 for details.).

Data sets are recorded using *EPU*. The microscope control system has a 70 TB NAS (Network-attached storage) unit for saving movies as they are obtained (we will refer to this NAS as *microscope-NAS*). This storage unit is shared through a Samba share and connected to the microscope private network using a 10 Gb connexion. *Scipion* is executed on a Linux server which has two 1 Gb NICs (network interface controllers) that connect it to the private network and the LAN (local area network) respectively (we will refer to this machine as *ScipionBox*). *ScipionBox* has 32 CPUs at 2.40 GHz, 62 Gb of RAM memory and 2 *Quadro M4000* GPUs. Finally, a second NAS with four 1 Gb NICs is linked to the private, LAN and WAN networks (we will refer to this second NAS as *Output-NAS*) so it can retrieve data from any computer and serve it to external users.

The data flow is as follows: (a) the microscope system stores movies in the *microscope-NAS*, (b) when executing movie-alignment protocols *ScipionBox* reads the movies from the *microscope-NAS* but does not store them locally, (c) *Output-NAS* has access to *microscope-NAS* and *ScipionBox* hard disks (d) *Output-NAS* has a collection of scripts than may be used to incrementally backup *Scipion* projects and the associated movies to a local or remote disk, or serve this information through the Internet, and (e) in parallel, periodically *ScipionBox* transfers to *HTML-Server* reports that may be checked by users or staff with an HTML browser (see a report example athttp://nolan.cnb.csic.es/scipionbox/lastHTMLReport/).

Although CNB staff try to discourage the use of external USB hard disks versus network as way of retrieving the raw and processed data, this is the preferred option for most users. USB 3.0 drives can theoretically handle data flows of 300 MB/s, but CNB experience is that, in this busy environment, many of them have trouble exceeding 50 MB/s. In most cases, 50 MB/s is enough to transfer the data before the following microscope turn-shift starts. Network users access to *Output-NAS* using a hardened common account. This account only allows rsync connections and make impossible to retrieve data if the name of the project to be downloaded is unknown.

#### 4.1.2 Additional software developments: *EMAdmin*

*EMAdmin* was born as a script that created a normalized tree of directories where movies acquired by the microscope could be stored, helped users to choose between a few predetermined image processing workflows and finally launched *Scipion*. At present it has grown in a client-server application that, in addition to the old script capabilities, has incorporated the following functions:

1. Transparent addition of new workflows. *Scipion* is able to export any executed workflow as a json file. *EMAdmin* can import this file and use it to create new predetermined workflows.
2. Automatic creation of pdf user’s report that contains microscope acquisition parameters and histograms summarizing data resolution and defocus.
3. The above mentioned information is stored in a database for further analysis. By default, the system plots the average resolution and astigmatism of the different acquisitions versus time.
4. Remote management. *EMAdmin* is a client server application, the client maybe executed in a machine different from the one that executes *Scipion*.
5. Automatic backup of the *Scipion* project.
6. *Scipion* is able to create live HTML reports on the image acquisition process. *EMAdmin* keeps track of all of them and facilitates the access to this information.

*EMAdmin* is free software available at github repository ( https://github.com/rmarabini/webservices/tree/master/EMadmin). The software can be freely downloaded and customized to meet the specific requirements of client organizations.

### 4.2 Case 2: *Scipion* at the Swedish National Cryo-EM Facility

The Swedish National Cryo-EM Facility has two nodes: at SciLifeLab in Stockholm and at Umeå University. SciLifeLab in Stockholm offers single-particle service with a Talos Arctica for sample optimisation and a Titan Krios for high-resolution data collection. The Umeå node offers cryo-ET with a Titan Krios and a Scios DualBeam SEM. Internally, an online booking system called *Booked Scheduler* centralizes the reservations of the microscopes and other instruments.

At the beginning of 2016, the Cryo-EM facility at SciLifeLab became one of the early adopters of *Scipion* streaming processing. Although the microscope operators were using home-made scripts to perform motion correction (with motioncor2) and CTF estimation (with Gctf), they recognized the importance of a more general framework. In particular, they were interested in the possibility of modifying easily the processing workflow as well as provide users and staff with graphical tools for data analysis and quick feedback regarding the data collection. Although initially the same setup script used at CNB was adopted, a new one was developed to fully satisfy the facility requirements. In the following sections we will briefly describe the computational infrastructure at the SciLifeLab node and the implementation of a Session Wizard to automate the initial setup while fetching information from other sources external to *Scipion*.

#### 4.2.1 Network setup and IT infrastructure

A storage and pre-processing server (the staging server) constitutes the core of the SciLifeLab computational infrastructure. This machine has roughly 200 TB of storage (4 ZFS RAIDZ2 pools with 11 HDDs each), 2 NVIDIA GeForce GTX 1070 GPUs, 2 Intel Xeon E5-2630v4 CPUs (10 cores, 2.2 GHz, HT), 384 GB RAM, and a dual-port 10 GbE network card. The storage pool is exported via NFS and Samba. The microscope computers can thus write data directly there and other machines can access it.

After processing, users may copy their data to external USB drives from two workstations in the microscope room that have access to the storage pool via NFS. There is also a dedicated download server which has access to the NFS export. This download server is accessible from the Internet and allows downloads via rsync (over SSH) or SFTP.

All these computers are connected to a 10 GbE network switch. The only machine accessible from the Internet is the data download server, which has a dual-port 10 GbE network adapter (one used for the internal network, one for the external network).

#### 4.2.2 Additional software developments: *Session Wizard*

**Fig. 2:**
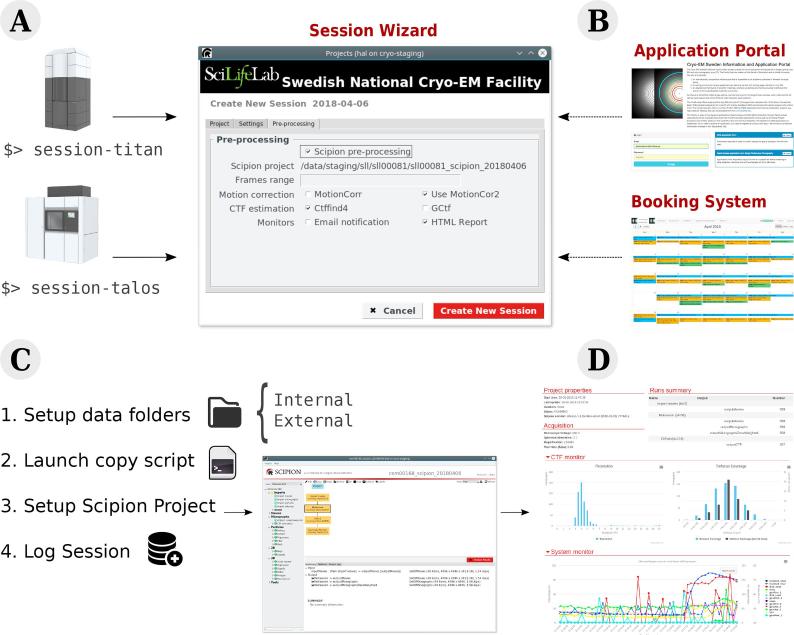
Overview of the Session Wizard developed at SciLifeLab. (A) The user triggers the wizard for any of the current microscopes. (B) The wizard retrieves project information from the *Application Portal* and reservation details from the *Booking System*. (C) The wizard creates data folders, executes the image copying script, configures the Scipion streaming project and logs the current session into a database. (D) The *Scipion* project is used to generate on the fly web reports and invoice documents later on.

In order to interface *Scipion* with the microscope infrastructure, an upgraded version of the original script developed at CNB was used. Unfortunately, it was difficult to reuse the existing code due to a different folder structure and, more important, a different approach to user and microscope management. To address the new set of requirements, a new wizard has been developed at SciLifeLab (see Fig. 2).

When a user starts a data collection, the wizard is executed using the command *session-titan* (or *session-talos*). The wizard determines which user has booked the microscope by fetching today’s booking information from *Booked Scheduler* through their web API, stores it, creates the processing environment and launches *Scipion*. The current implementation of the wizard stores each session in a simple database file. This database, together with extra information from the Booking System and an Application Portal, is used to generate invoices and reports for a given period.

The described session wizard can be found at  https://github.com/delarosatrevin/scipion-session. The software can be freely downloaded and customized to meet the specific requirements of client organizations. *Booked Scheduler* is available at ( https://www.bookedscheduler.com/).

### 4.3 Case 3: *Scipion* at eBIC (Diamond Light Source)

Diamond Light Source is the UK National Synchrotron User Facility. As such, it provides access to large-scale experimental facilities to the UK and worldwide scientific communities to conduct experiments that are not possible in their home laboratories. As a consequence of this national role, Diamond has significant experience at managing many visiting scientists and user experiments, and the associated data and post-processing. Recently, the electron Bio-Imaging Centre (eBIC) has been set up at Diamond (Clare et al., 2017). The centre houses four Thermo Fischer Scientific Titan Krios, a Talos Arctica and a Scios dual-beam. All of the transmission electron microscopes are equipped with counting-mode direct electron detectors and Volta phase plates.

#### 4.3.1 Network setup and IT infrastructure

Diamond has thousands of users every year, both academic and commercial, and it is vital that their data is kept secure and confidential. A user management system has been developed to allocate each user with a unique federal identity (ID), and to associate this ID with the experiments in which the user partakes in. Users may access their data through SynchWeb which fronts ISPyB, a Laboratory Information Management System combining sample tracking and experiment reporting (Delageniere et al., 2011, Fisher et al., 2015).

Data is collected onto a local machine and then immediately moved via 10Gbps Ethernet to the associated visit directory in the centralized storage, a multi Petabyte GPFS high performance parallel file system. Once the raw data is present on the GPFS system, high speed interconnects to the central cluster enable rapid data processing.

The complexity of Diamond IT infrastructure requires to integrate many heterogeneous systems. In this environment a major problem is to achieve interaction and data exchange between diverse applications. One of the solutions to this problem has been to implement *indirect communication* through a message queuing system called *ActiveQ*. A message queue is a mechanism that allows a sender process and a receiver process to exchange information; instead of connecting directly, the sender posts messages in a queue and the receiver, when ready, takes these messages from the queue and process them appropriately.

### 4.4 Additional software developments: ISPyB monitor, *Scipion* Headless

From the image processing point of view, in Diamond, a typical cryoEM session starts by launching a custom made script that allows users to specify values pertinent to their experiment, namely the session ID (or visit number), which microscope they are using, and several other key data collection parameters. Once set, a file describing a *Scipion* workflow is created and then saved to disk. A daemon that is running on the microscope control server notices the file creation and sends a “file creation” message to *ActiveMQ*.

Another process listening to *ActiveMQ* then launches *Scipion* from this workflow and creates a running “headless” project. *Scipion* headless mode has been developed specifically for eBIC and allows *Scipion* to be executed without graphical user interface in large clusters with no graphical board.

Unlike CNB and SciLifeLab cryo-EM services, where the Scipion default monitor system displays the progress of the automatic image processing, in Diamond, the electronic notebook ISPyB is the natural framework to record and show metadata about the different experiments. A new monitor has thus been developed to allow *Scipion* to directly interface with ISPyB using the ISPyB python API. This setup provides the users with a web interface showing general metadata about their experiment, as well as real-time updates on steps of the process and relevant output, such as motion corrected images, drift values, CTF images, etc.

At present, most of the developments described in this subsection are available in a developer’s branch at  https://github.com/I2PC/scipion/tree/release-1.1-headless-devel. Some of them (e.g. the monitor protocol) will be integrated in the main *Scipion* repository while the more Diamond specific software will be published in an independent repository. *Scipion* headless mode is already integrated in the main *Scipion* branch.

## 5 Future developments and usage statistics

### 5.1 Future developments

Currently, *Scipion* includes the appropriate code (python wrappers) to talk to the integrated EM packages (Xmipp, Eman, Relion, …) at the time of the release. Therefore, an update of any of the integrated EM packages immediately after the *Scipion* release will not be available until next *Scipion* release (we are aiming for one major release per year). To decouple EM-package releases from *Scipion* releases, we are working on making the wrappers for EM packages independent from *Scipion*. Plans for ensuring this independence include: (1) wrapper installation through pip ( https://pip.pypa.io/en/stable/), (2) reimplementation of the wrappers as plug-ins that will be automatically detected and added to the application menu and (3) develop of standalone IO and visualization libraries that make *Scipion* fully independent from other EM packages.

Additionally, in collaboration with the Xmipp team, we are pushing the streaming workflow with the aim of obtaining a first initial volume. We are also adding structure modeling capabilities, by integrating some functionality from programs such as Coot (Emsley et al., 2010), Refmac (Murshudov et al., 1997), etc.

As part of the EOSCPilot project ( https://eoscpilot.eu/cryoem), we have improved how *Scipion* exports workflows in order to ease reporting of the work done. In this way we want to make the data produced by *Scipion* more compliant with the FAIR guidelines given by the EUROPEAN COMMISSION Directorate-General for Research and Innovation (2016). This workflow file could go with the raw data acquired by CryoEM facilities and deposited at common EM databases such as EMPIAR or EMDB (Patwardhan and Lawson, 2016). To facilitate the visualization of the workflow file in any of these databases, we are developing a webcomponent (*Scipion workflow viewer*) that will easily allow these repositories to visualize the workflow on their web pages ( https://github.com/I2PC/web-workflow-viewer). Finally, we are implementing a workflow repository. This repository will contain ready-to-use workflows that support a range of use cases. A preliminary beta version of the workflows repository is available at  http://workflows.scipion.i2pc.es/.

### 5.2 Usage statistics

In the context of free and open source projects it is difficult to create usage statistics that are accurate. But certainly, if we want *Scipion* to succeed, we need to know how frequent the different protocols are used. This is the reason why we have recently developed our *Scipion Usage Data Collector* that monitors -if activated by the user- the usage of the different protocols and send -via HTTP protocol- the information to the developer’s team. This information is anonymous and cannot be used to identify the original computer in which *Scipion* has been executed.

Using the HTTP header it is possible to guess the country in which *Scipion* has been installed. With these information we have created the usage map and table available at URLs  http://scipion.i2pc.es/report_protocols/scipionUsage/ and  http://scipion.i2pc.es/report_protocols/protocolTable/. The data shows “global” *Scipion* usage rather than “facility” usage but there is a one to one relationship between the countries with highest number of *Scipion* projects and the countries with *Scipion* based EM facilities. The only exception to the rule is Canada where two of the original *Scipion* developers are setting up a new facility at McGill University.

Note that, in a effort to report real use and skip test projects, the number of projects in the map refers to the number of non empty *Scipion* projects updated more than once. That is, *Scipion* projects that have been opened in at least two different days. Another constraint is that developers’ computers are filtered out.

## 6 Conclusions

The new generation of high-end cryo electron microscopes and direct electron detectors are behind the recent increase in cryo-EM popularity. However, the run and maintenance costs of these new microscopes are high and can only be justified by keeping the microscopes up and running 24 h a day. Therefore, microscopes tend to belong to centralized facilities where users are allocated microscope time (shifts) to perform a variety of tasks. Available shifts are a scarce commodity and therefore users (and staff) need to be able to monitor the quality of the acquisition process and obtain, as soon as possible, a first glimpse of the reconstructed specimen in order to provide an early detection of any issue.

Workflow systems have been explored and used extensively in bioinformatics but they have raised little interest in the cryo-EM community. *Scipion* has been designed following a strict workflow paradigm. This approach is specially useful for tasks that need to go from a given start (movies) to a defined end (3D map), but where there are many different paths (algorithms) and steps to get there.

Workflows implement pipelines and describe them as a set of interconnected steps. In a workflow approach, users (including microscope staff) can reasonably be expected to modify the paths and incorporate new steps using a GUI. Alternate solutions as scripts do not clearly separate each step definition from the connections between the different steps and, for no programmers, are scary and difficult to modify. The *Scipion* workflow approach provides provenance, that is, for any created object (micrograph, CTFs, class, etc) *Scipion* stores metadata such as which programs, which parameters, and which exact path was followed to create any output data. In this way, real tracking and reproducibility are provided.

*Scipion* stream capabilities can handle large and continuously produced volumes of data. The benefits of streaming are clear: (1) it helps to process data as soon as it is generated, providing almost instant results on data quality; (2) it saves user processing time and resources at the user home institution since part of the processing is done while data is being acquired; and (3) it further reduces the overall experimental time by allowing processing the data while it is transferred to the final user laboratory.

*Scipion* has been deployed in cloud and there is a public image, with all the required preinstalled cryoEM software, available at Amazon WebServices EC2 and EGI federated clouds. This image makes use of *Scipion* streaming capabilities, that is, they are able to process data as soon as it has been transfered to an Amazon storage unit, and can be used to handle production peaks.

*Scipion* is provided freely as open source software. Online documentation describing *Scipion* download and installation is available at  http://scipion.cnb.csic.es/m/download_form/ and  https://github.com/I2PC/scipion/wiki/How-to-Install, respectively. Cloud installation instructions are available at  https://github.com/I2PC/scipion/wiki/Scipion-in-the-Cloud. Other public software mentioned in this article as *EMAdmin*, *wizard session* and the different scripts used at eBIC are available at  https://github.com/rmarabini/webservices/tree/master/EMadmin,https://github.com/delarosatrevin/scipion-session and  https://github.com/I2PC/scipion/tree/release-1.1-headless-devel respectively. *Scipion* download statistics, usage map and protocol usage ranking are available at  http://scipion.i2pc.es/downloadstats,  http://scipion.i2pc.es/report_protocols/scipionUsage/ and  http://scipion.i2pc.es/report_protocols/protocolTable/ respectively.

For more information about how to install *Scipion* or suggestions to improve it, contact us at scipion@cnb.csic.es.
